# Green tea extract and hydroxyl-chloroquine combination enhances apoptosis in A549 non-small cell lung cancer cells

**DOI:** 10.6026/97320630019860

**Published:** 2023-08-31

**Authors:** Tanmayi Atchuta Sri Lakshmi K, Rahul Chidurala, Suresh Parepalli, Karthik VP

**Affiliations:** SRMC and RI, Sri Ramachandra Institute of Higher Education and Research-DU, Porur, Chennai-600116, Tamil Nadu, India; Department of Pharmacology, SRMC and RI, Sri Ramachandra Institute of Higher Education and Research-DU, Porur, Chennai-600116, Tamil Nadu, India

**Keywords:** Green Tea, MTT Assay, non-small lung cancer cell, nslcc a549 cells, fluorescence, cytotoxicity

## Abstract

Polyphenols, including catechins from green tea extract, have long been known for their potential anti-tumour activities. However, the precise mechanisms underlying their actions remain unclear. This study aimed to investigate the effects of green tea extract on A549 cells, a type of non-small lung cancer cells. A549 cells treated with green tea extract (GTE) were examined using an inverted light microscope and a fluorescence microscope. Cell sensitivity was evaluated using the MTT assay, while cell death was assessed using the Tali image-based cytometer. Ultra structural changes were observed using a transmission electron microscope. The findings suggested that even at the highest dose tested (150 µM), GTE did not exhibit toxic effects on A549 cells. Likewise, treatment with GTE resulted in a minimal, dose-dependent increase in the population of apoptotic cells. However, the analysis of cell structures using light and electron microscopy revealed an enhanced accumulation of vacuole-like structures in response to GTE. Moreover, under the fluorescence microscope, an increase in acidic vesicular organelles and the formation of LC3-II puncta were observed following GTE treatment. Assessment of autophagy function indicated that GTE-induced autophagy may serve as a self-protective mechanism against cytotoxicity, as blocking autophagy with bafilomycin A1 reduced cell viability and enhanced necrotic cell death in response to GTE treatment. In summary, our results demonstrate that A549 cells are insensitive to both low and high concentrations of green tea extract, likely due to the induction of cytoprotective autophagy. These findings suggest that the potential utility of GTE in lung cancer therapy may lie in its synergistic combinations with drugs or small molecules that target autophagy, rather than as a standalone therapy.

## Background:

Tea is one of the most popular beverages in the world and is produced from the leaves of *Camellia sinensis*, but the type of tea depends on the process of its manufacture [1]. All types of tea contain active polyphenols, commonly known as catechins [2]. Green tea contains polyphenols such as epigallocatechin, epigallocatechin-3-gallate (EGCG), epicatechin, andepicatechin-3-gallate, but EGCG is the most abundant and possibly the most bioactive constituents [3]. Many epidemiological studies have shown that green tea may help to protect against cancer development [4]. The EGCG may act proactively against the oesophagal, lung, prostate, and stomach, intestine, breast, and colon carcinogenesis process [5]. Green tea extract (GTE) consumption is also believed to protect against the augment of atherosclerosis and coronary heart disease, high blood cholesterol concentrations, and high blood pressure [6]. It is known that the biological activities of EGCG are associated with anti-inflammatory and antioxidant properties [7]. Furthermore, the study on the mechanisms involved in the chemoprevention of green tea has revealed its impact on the modulation of signal transduction pathways that lead to the inhibition of cell proliferation and transformation, inhibition of tumour invasion and angiogenesis and induction of cell death [8]. The most common types of cell death which are induced by EGCG are apoptosis and autophagy. Additionally, the crosstalk between autophagy and apoptosis has recently been explored in various researches [9]. The first type of cell death is characterized by chromatin condensation, nuclear fragmentation and blebbing formation. EGCG induces apoptosis and inhibits proliferation in numerous cancer cell lines, inter alia in gastric cancer cells and human breast cancer cells [10]. It has also been shown that GTE enhances the cytostatic effect of conventional anticancer drugs in chemo-resistant cancer cells. The synergistic apoptotic effect of panaxadiol and EGCG in human colorectal cancer cells [11,12]. Autophagy, the second type of cell death that has been observed in cells treated with GTE, is characterized by the formation of autophagosomes and auto phagolysosomes containing unnecessary or dysfunctional cellular components and unused proteins [13]. In the literature, three types of autophagy are described micro-autophagy, chaperone-mediated autophagy and macro autophagic but the best described and understood is macro autophagy. Autophagy can be stimulated by a lack of nutrients, reactive oxygen species, hypoxia, and toll-like receptor agonists. It is known to be involved in inflammation, ageing, cancer, neurodegeneration, cardiac myopathies, and infections [14]. Generally, autophagy inhibits apoptosis, but in special cases, autophagy can induce apoptosis. Additionally, massive autophagy has been shown to promote autophagic cell death, also known as type II Programmed cell death [15]. The present study aimed to determine the effect of GTE and hydroxyl chloroquine (HCQ) as well as their combination effects on human non-small cell lung carcinoma cells (A549).

##  Materials and Methods:

## Cell lines:

A549 cell line was obtained from National Centre for Cell Sciences, Pune (NCCS).

## Cell culture and treatment:

A549 cells (non-small lung cancer cell line; NSCLC) were grown in a monolayer at 37°C in a humidified CO2 incubator (5% CO2) in Dulbecco's Modified Eagle's Medium (DMEM, Lonza) with the addition of 10% FBS (fetal bovine serum; Gibco) and 50 µg/ml of gentamycin (Sigma-Aldrich). A stock solution of green tea extract (GTE, Santa Cruz) was prepared in distilled water and stored at low temperatures until use. The required concentrations of GTE (25, 50, 100, 150 µM) were added to cells for 24 h for MTT assay. HCQ was used at dilutions of 1:1 to 1:16. For other experiments, the non-small lung cancer cells were treated with GTE at final concentrations of 25, 50 and 150 µM for 24 h. Control cells were incubated under identical conditions without the addition of green tea extract. Furthermore, to inhibit autophagy, the cells were pretreated for 8 h with bafilomycin A1 (Baf A1) at a concentration of 100 nM followed by 24 h incubation with GTE. The A549 cells were observed using an inverted microscope (Nikon). All chemicals and reagents used in this study were obtained from Sigma Aldrich, India

## In Vitro assay for anti-cancer activity (MTT assay):

Cells (1 x 105/well) were plated in 24-well plates and incubated at 370C with 5% CO2 condition. After the cell reaches the confluence, the various concentrations of the samples were added and incubated for 24hrs. After incubation, the sample was removed from the well and washed with phosphate-buffered saline (pH 7.4) or DMEM without serum. 100µl/well (5mg/ml) of 0.5% 3-(4,5-dimethyl-2-thiazolyl)-2,5-diphenyl--tetrazolium bromide (MTT) was added and incubated for 4 hours. After incubation, 1 ml of DMSO was added to all the wells. The absorbance at 570nm was measured with UV- Spectrophotometer using DMSO as the blank. Measurements were performed and the concentration required for a 50% inhibition (IC50) was determined graphically [16]. The % cell viability was calculated using the following formula:

% Cell viability = A570 of treated cells / A570 of control cells x 100

Graphs are plotted using the % of Cell Viability at the Y-axis and the concentration of the sample at the X-axis. Cell control and sample control are included in each assay to compare the full cell viability assessments.

## Fluorescent staining Technique:

A549 cells were seeded (one lakh cells per ml) on a cover slip placed in a 6-well plate and incubated for 24 hours. After incubation, the monolayer of cells was treated with IC50 concentration of the sample and incubated for 24 hours.

The treated cells were washed with sterile PBS. 2% glutaraldehyde and ethanol were used to fix the cells. 0.1 ml of Propidium Iodide stain was added to the cells and left for 15 to 30 minutes in the dark. The coverslip containing the stained cells was removed from the 6-well plate and placed on a clean grease-free glass slide and fluorescent images were taken.

## Statistical analysis:

All the experiments were performed independently in triplicate. The results are provided as the mean ± the standard deviation (SD). For comparisons among multiple groups, an analysis of variance was used. SPSS 16.0(IBM Corp., Armonk, NY, USA) was used to perform statistical analysis. Statistical significance was accepted at P<0.05.

## Results and Discussion:

## Effect of Green tea on cell viability of A549 cells

**Figure 1:**
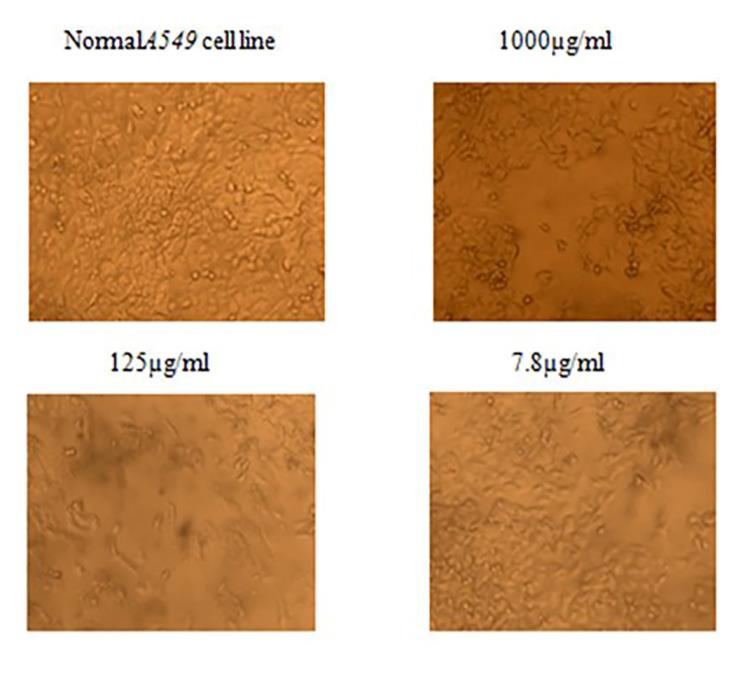
Anticancer effect of Green Tea Extract on A549 cell line

**Figure 2:**
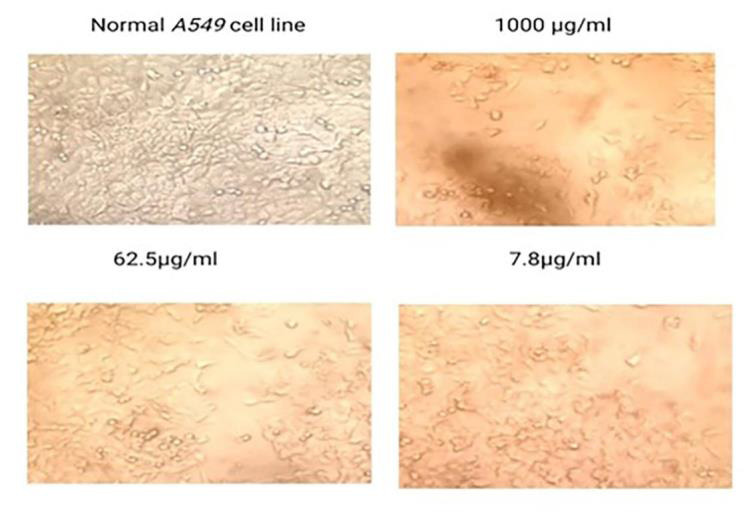
Anticancer effect of Hydroxychloroquine on A549 cell line

The results of the MTT assay were used to evaluate the viability of cells and showed the treatment with green tea at 1000, 500, 250, 125, 62.5, 31.2, 15.6, and 7.8 concentrations significantly inhibited A549 cell proliferation. Increasing concentration significantly decreased cell viability. The IC50 values revealed effective decrease in cell viability with combination treatment of GTE and HCQ (Table 1, Table 2,Table 3). The study assessed the sensitivity of the A549 non-small lung cancer cell line to the green tea extract. For this purpose, we treated A549 cells with GTE at concentrations of 25, 50 and 150 µM for 24 h and MTT colourimetric assays were performed. The obtained data suggested that GTE, even at the highest dose, was not toxic to A549 cells. As shown in Figure 1, compared to the control populations, 97.06%, 94.31% and 93.56% of A549 cells survived after exposure to 25, 50 and 150 µM GTE, respectively. Next, we performed an apoptosis analysis of A549 cells in response to GTE treatment. The cells were double-stained with Annexin V and PI and the percentage of cells in each population (viable Annexin V-/PI-; early apoptotic Annexin V+/PI-; late apoptotic Annexin V+/PI+; necrotic Annexin V-/PI+) was evaluated using an image-based cytometer. The sum of the early and late apoptotic cells represented the total apoptosis. As can be seen in Figure 2B, the treatment with 25, 50 and 150µM GTE for 24 h resulted in a small but statistically significant increase in the population of apoptotic cells from 0.075% to 0.45-3.2%. Moreover, there were no statistically significant differences in the percentage of necrotic cells after the exposure of A549 cells to green tea extract when compared to the control, except for the highest dose of GTE plus Baf A1 and Baf A1 alone (Figure 2).

**Figure 3:**
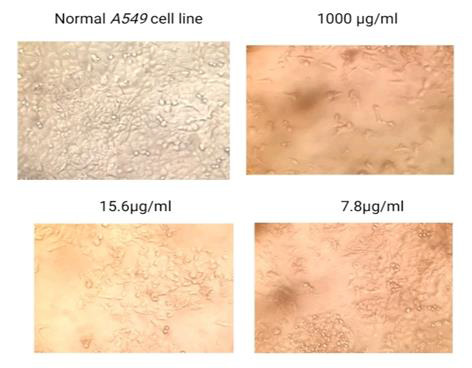
Anticancer effect of Green Tea Extract + Hydroxy chloroquine on A549 cell line

To study the effect of green tea extract on A549 cells we used an inverted microscope to analyze the cell morphology. After the treatment with GTE, a lot of vacuole-like structures in the cytoplasm of A549 cells were seen (Figure 3). The severity of these changes appeared to be directly related to the GTE doses. To make sure that the observed vacuole-like structures were in fact due to the induction of autophagy, we used a specific autophagy inhibitor, bafilomycin A1, which prevents the maturation of autophagic vacuoles by inhibiting the fusion between autophagosomes and lysosomes [17]. The Baf A1 pretreatment almost completely abolished the GTE-induced vacuole-like structure formation in A549 cells is seen and thus confirming that those changes were associated with autophagy.

**Figure 4:**
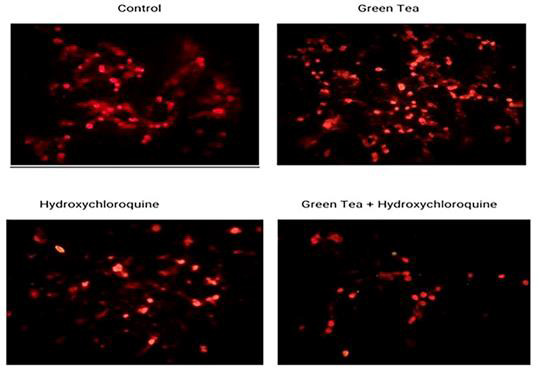
Fluorescent staining

This finding, together with the observation of high viability of A549 cells after treatment with GTE, led us to presume that GTE might induce autophagy in these cells because many previous studies have shown that autophagy i) associated with the formation of auto phagosomes and auto phagolysosomes and ii) facilitates the resistance of tumour cells against chemotherapy and radiation [18]. To further confirm whether GTE could trigger autophagy, we studied the ultrastructure of GTE-treated A549 cells in the transmission electron microscope, since TEM has been considered as a gold standard to observe autophagic vacuoles [19]. As we expected, GTE promoted the accumulation of autophagic vacuoles, which exhibited auto lysosomal and/or autophagosome characteristics (Figure 4). Histopathological analysis showed that numerous empty vacuoles, as well as vacuoles filled with remnants of organelles, were observed (Figure 2). Another characteristic ultra-structural feature of autophagy was noticed: swollen mitochondria devoid of cristae.

To further study the incidence of autophagy, we examined the impact of GTE on the occurrence of acidic vesicular organelles (AVOs) by staining cancer cells with acridine orange (AO). The detection of AVOs, which include autophagic vacuoles and lysosomes, is a standard method for monitoring autophagy [20]. AO is a fluorescent weak base that accumulates in acidic spaces, such as AVOs, and emits bright red fluorescence. The intensity of the red fluorescence is therefore proportional to the degree of acidity and the volume of acidic vesicular organelles [21]. As shown in Figure 3, in A549 cells GTE promoted the formation of AVOs in a dose-dependent manner, the process of which was almost completely blocked by pretreating with Baf A1. We have also examined the effect of GTE on the intensity and pattern of LC3-II (a specific marker of autophagy) immuno staining.

The fluorescence microscopy analysis revealed the punctate staining pattern and the increased fluorescence intensity of this protein in the A549 cells exposed to GTE (Figure 4), in comparison to the diffuse staining pattern and low fluorescence intensity of LC3-II in control cells. Such an effect of GTE was dose-dependent. Pretreatment with Baf A1 resulted in further accumulation of LC3-II in A549 cells (Figure 4). Collectively, our results suggested that GTE, especially at higher doses, triggered autophagy in A549 cells. Similar observations have been presented by Kim et al. (2013), who observed an increase in the formation of LC3-II and autophagosomes following treatment with EGCG of primary bovine aortic endothelial cells (BAEC) [22]. As mentioned above, the high viability of A549 cells after treatment with GTE allowed us to assume that there must be some mechanism that protects these cells against GTE-induced cytotoxicity.

Even though autophagy has recently been proposed to be a type of cell death, the primary role of autophagy is to protect cells under stressful conditions such as starvation, hypoxia, and drug treatment as well [23]. Satoh et al. (2013) studied the effect of EGCG on five human mesothelioma cell lines and revealed that the inhibition of autophagy by chloroquine (CQ) enhanced the EGCG-induced cell death [24]. Therefore, we presumed that GTE-induced autophagy may have a cytoprotective effect on A549 cells. To test this hypothesis, we pretreated A549 cells with Baf A1 for 8 h and then incubated them for 24 h with GTE. After that, the MTT assays were performed. As shown in figure 1, autophagy blockage significantly decreased the viability of A549 cells from 95.10% (Baf A1 alone) to 72.43% (Baf A1 plus 150µM GTE), confirming our assumptions. Since it has been shown that the inhibition of the cytoprotective role of autophagy may enhance the apoptotic response to anticancer drugs, we repeated the Annexin V/PI assays using Baf A1[24]. Surprisingly, we did not observe an increase in the percentage of apoptotic cells in the populations co-treated with Baf A1 and GTE at a concentration of 150 µM in comparison to cells exposed to Baf A1 alone (Figure 2). Therefore, we assume that autophagy and apoptosis in A549 cells treated with GTE are independent processes. Indeed, there is currently accumulating evidence that autophagy and apoptosis can act either as partners to induce cell death or autophagy may act as an antagonist to inhibit apoptosis, thereby promoting cell survival, or apoptosis and autophagy may occur independently of each other [25, 26]. However, the blockade of autophagy by Baf A1 resulted in increased necrotic cell death in response to GTE (from 1.98% and 4.62% in the control and Baf A1-treated cells, respectively, to 15.25% in co-treated cells), further suggesting that GTE-induced autophagy plays a cytoprotective role against its cytotoxic effect.

To sum up, the results presented here revealed that A549 cells are insensitive to both low and high concentrations of green tea extract, probably due to the induction of cytoprotective autophagy [27]. This data confirms a common observation that dietary polyphenols alone fail to affect the growth of chemo-resistant cancer cells. Therefore, the potential of these compounds as anticancer agents lies in combination therapies with conventional cytostatic drugs to decrease their effective doses and/or enhance their efficacy. It is important to clarify, in vitro and in vivo, whether autophagy inhibitors such as chloroquine, which is currently under clinical trials, could act synergistically with GTE to produce a growth-inhibitory effect on chemo-resistant cancer cells [28].

## Conclusion:

It is concluded that the study employed various techniques, including the MTT assay, cytometry, and fluorescent techniques, to assess the potential anti-cancer effects of green tea extract. The results of the study indicated that green tea extract exhibited significant cytotoxic effects on the A549 cell line. The MTT assay revealed a dose-dependent decrease in cell viability, suggesting that the extract had a direct impact on the cancer cells. Furthermore, cytometry analysis demonstrated an increase in apoptotic cells, indicating that green tea extract induced programmed cell death in the A549 cells. Fluorescent techniques were utilized to gain further insights into the mechanisms of action of green tea extract. These techniques allowed the researchers to visualize and quantify specific cellular processes, such as DNA fragmentation and changes in mitochondrial membrane potential. The findings indicated that green tea extract caused DNA damage and disrupted mitochondrial function in the A549 cells, further contributing to their demise. Overall, this study provides compelling evidence for the cytotoxic effects of green tea extract on non-small cell lung carcinoma cells. The use of multiple techniques strengthened the validity of the results and provided a comprehensive understanding of the underlying mechanisms. These findings support the potential use of green tea extract as a natural therapeutic adjuvant for the treatment of non-small cell lung carcinoma. Further studies are warranted to explore its efficacy and safety in clinical settings and to elucidate the specific bioactive compounds responsible for its anti-cancer effects.

## Competing interest:

The authors declare that they have no competing interests.

## Funding:

This research received specific grant from Chancellor's Summer Research Fellowship for UG students/2020

## Figures and Tables

**Table 1:** Anticancer effect of Green Tea ExtractonA549 cell line

**S. No**	**Concentration (µg/ml)**	**Dilutions**	**Absorbance (O.D)**	**Cell viability (%)**
1	1000	Neat	0.455	29.44
2	500	1:01	0.581	37.6
3	250	1:02	0.707	45.76
4	125	1:04	0.832	53.85
5	62.5	1:08	0.957	61.94
6	31.2	1:16	1.082	70.03
7	15.6	1:32	1.207	78.12
8	7.8	1:64	1.332	86.21
9	Cell control	-	1.545	100

**Table 2:** Anticancer effect of Hydroxychloroquine on A549 cell line

**S.No**	**Concentration (µg/ml)**	**Dilutions**	**Absorbance (O.D)**	**Cell viability (%)**
1	1000	Neat	0.341	22.07
2	500	1:01	0.453	29.32
3	250	1:02	0.567	36.69
4	125	1:04	0.68	44.04
5	62.5	1:08	0.794	51.39
6	31.2	1:16	0.908	58.77
7	15.6	1:32	1.021	66.08
8	7.8	1:64	1.134	73.39
9	Cell control	-	1.545	100

**Table 3:** Anticancer effect of Green Tea Extract + Hydroxychloroquine on A549 cell line

**S.No**	**Concentration (µg/ml)**	**Dilutions**	**Absorbance (O.D)**	**Cell viability (%)**
1	1000	Neat	0.122	7.89
2	500	1:01	0.235	15.21
3	250	1:02	0.343	22.2
4	125	1:04	0.457	29.57
5	62.5	1:08	0.566	36.63
6	31.2	1:16	0.674	43.62
7	15.6	1:32	0.761	49.25
8	7.8	1:64	0.876	56.69
9	Cell control	-	1.545	100
